# Clinical Characteristics and Pathogen Spectrum of Male Genital Fungal Infections in Nanchang Area, South China

**DOI:** 10.1007/s11046-024-00839-0

**Published:** 2024-04-16

**Authors:** Yun Jin, Yangmin Gao, Yunpeng Luo, XiaoHua Tao, Qing Jiang, Xinyi Fan, Rui Xu, Hua Qian, Xiaoguang Li, Zhijun Zhou

**Affiliations:** 1 Dermatology Hospital of Jiangxi Province, Jiangxi Provincial Clinical Research Center for Skin Diseases, Candidate Branch of National Clinical Research Center for Skin Diseases, 388 Yingbinbei Road, Nanchang, 330001 China; 2 School of Public Health and Laboratory Medicine, Hunan University of Medicine, 492 Jinxi South Road, Huaihua, 418000 China; 3 Department of Laboratory Medicine, Chronic Disease Research Center, Medical College, Dalian University, Dalian, China

**Keywords:** Fungal infection, Male genitalia, Etiology, *Trichophyton rubrum*

## Abstract

The cutaneous fungal infections in male genitalia are relatively rare, and often present with various atypical clinical symptoms. It was mainly reported in a small number of case reports, while data with large number of patients were rarely reported. In this study, we reported 79 male patients with cutaneous fungal infections on scrotum or penis. The fungal infections were confirmed by microscopic examination directly and fungus culture. Clinical characteristics and predisposing factors were also collected. Of these 79 patients, 72 has lesions on scrotum, 5 on penis and 2 on both scrotum and penis. *Trichophyton (T.) rubrum* is the most common pathogen, found in 50 (67.6%) patients, which presented diverse clinical manifestation such as majorly erythematous, dry diffused scaly lesions without a clear border, slightly powdery and scutular scalings. *Candida (C.) albicans* is the secondly common pathogen, found in 21 (28.4%) patients, which also presented diverse lesions such as erythematous with dry whitish scaly lesions and erythematous erosion. The predisposing factors mainly included concomitant fungal infections on sites other than genitalia, especially inguinal region (tinea cruris), application of corticosteroid and high moisture. In conclusion, cutaneous fungal infections in male genitalia could be caused by different fungi, showed atypical or mild clinical appearances in most cases and might be a fungus reservoir, emphasizing the necessity to timely perform the fungi examinations and corresponding therapy.

## Introduction

The cutaneous fungal infections on male genitalia including scrotum and penis are mainly caused by dermatophytes or yeasts [1]. Most of the studies with large data on superficial fungal infections have higher isolation rates of tinea cruris, but very less data of cutaneous fungal infections on scrotum or penis [2, 3]. Compare to other cutaneous sites, the fungal infection of male genitalia was relatively rare reported, most of which were a small number of cases [4–13]. By the routine detection of fungal infections, we often found that some patients were positive for fungal infections in scrapings from scrotum by direct microscope, but showed no clear clinical features of fungal infections. In addition, the dominant etiology of fungal infections on male genitalia varied in different regions [14], and most of clinical manifestations were also different from tinea corporis and tinea cruris [4, 5, 9–12]. Therefore, it is necessary to perform comprehensive analyses of cutaneous fungal infections on male genitalia.

In the present study, clinical features, identified pathogens and predisposing factors of 79 male patients with cutaneous fungal infections on scrotum or penis, were collected and analyzed. The results indicated that cutaneous fungal infections in male genitalia could be caused by different fungi and showed atypical or mild clinical appearances.

## Material and Methods

### Patients

A prospectively observational study was carried out in outpatients from Dermatology Hospital of Jiangxi Province in Nanchang, Jiangxi province in southern China from January 2019 to October 2021. Total 93 cases of patients visited our hospital for mycology examination and the scrapings from their scrotum and/or penis were positive by direct microscopy, 79 of these patients agreed to perform further fungal culture and offered medical questionnaire survey. Clinical data including age, date visiting, time from disease onset to diagnosis, clinical presentation, cutaneous pruritus and different risk factors such as concurrent fungal infections, occlusive clothing, systemic diseases, topical corticosteroid agents, pet contact and high moisture were also collected.

### Microscopic Examination and Fungal Culture

Skin scrapings of the scrotum and/or penis were collected for mycological examination consisted of direct microscopy by 10% potassium hydroxide (KOH) or mycotic fluorescent solution and culture for causative agents by four tubes of Sabouraud dextrose agar (SDA), two with added cycloheximide and chloramphenicol, another two only with added chloramphenicol. The cultures were incubated at 28 °C for up to 2 weeks and the cultural results were observed every 2 days; the preliminary identification of the colonies depended on their macroscopic and microscopic characteristics. The DNA extraction was performed using Fungal DNA extraction kit from Omega Bio-Tek (Fungal DNA Kit D3390, Sangon Biotech corporation, Shanghai, China). PCR (Polymerase Chain Reaction) was amplified by using the primers ITS1 (5′-TCCGTAGGTGAACCTGCGG-3′) and ITS4 (5′-TCCTCCGCTTATTGATATGC-3′). PCR samples were sended to Sangon Biotech Corporation and sequence results of the strains were obtained. Sequences were compared with the NCBI (National Center for Biotechnology Information) database, and a similarity more than 99% was considered as successful identification at the species level.

### Statistical Analyses

Statistical analysis was performed with SPSS software (version 24.0; SPSS Inc. Chicago, IL, USA). The mean and SD are presented for continuous variables. Categorical variables are reported as the number of patients in each group and their percentage from the available data. The chi-square test was employed to analyze the differences of parameters between groups.

## Results

### Study Population

Total 79 male patients with genital fungal infections were collected by positive hyphae under the microscope in the present study. The median age was 36 years (range from 12 to 86 years), and 39 (49.4%) patients belonged to the age group 21–40 years, followed by 17 (21.5%) in 41–60 years age group. The mean onset time of infection before visiting our hospital was 14.93 months (range, 3 days to 8 years). In the 79 patients, scrotal lesions were found in 72 (91.2%) patients, penis lesions in 5 (6.3%) patients, and both scrotal and penis lesions in 2 (2.5%) patients.

### Identification of Pathogenic Fungi

The causative pathogen species were identified by positive cultures in 74 (93.7%) patients. The distribution of culture positive pathogens was shown in Table 1. The identification results and GenBank numbers for all genital isolates were summarized in Table S1. *T. rubrum* was the most frequent pathogen isolated, positive in 50 (67.6%) patients, followed by *C. albicans*, in 21 (28.4%) patients, *Nannizzia (N.) gypsea* in 2 (2.7%) patients and *Epidermophyton (E.) floccosum* in 1 (1.3%) patient.

**Table 1:** The distribution of culture positive pathogens in 74 cases

Infection sites	Etiology, case number				Total (%)
	*T. rubrum*	*C. albicans*	*N. gypsea*	*E. floccosum*	
Scrotum	45	19	2	1	67(90.5)
Penis	4	1	0	0	5(6.8)
Both scrotum and penis	1	1	0	0	2(2.7)
Total (%)	50(67.6)	21(28.4)	2(2.7)	1(1.3)	74(100)

### Clinical Characteristics

**Fig. 1:**
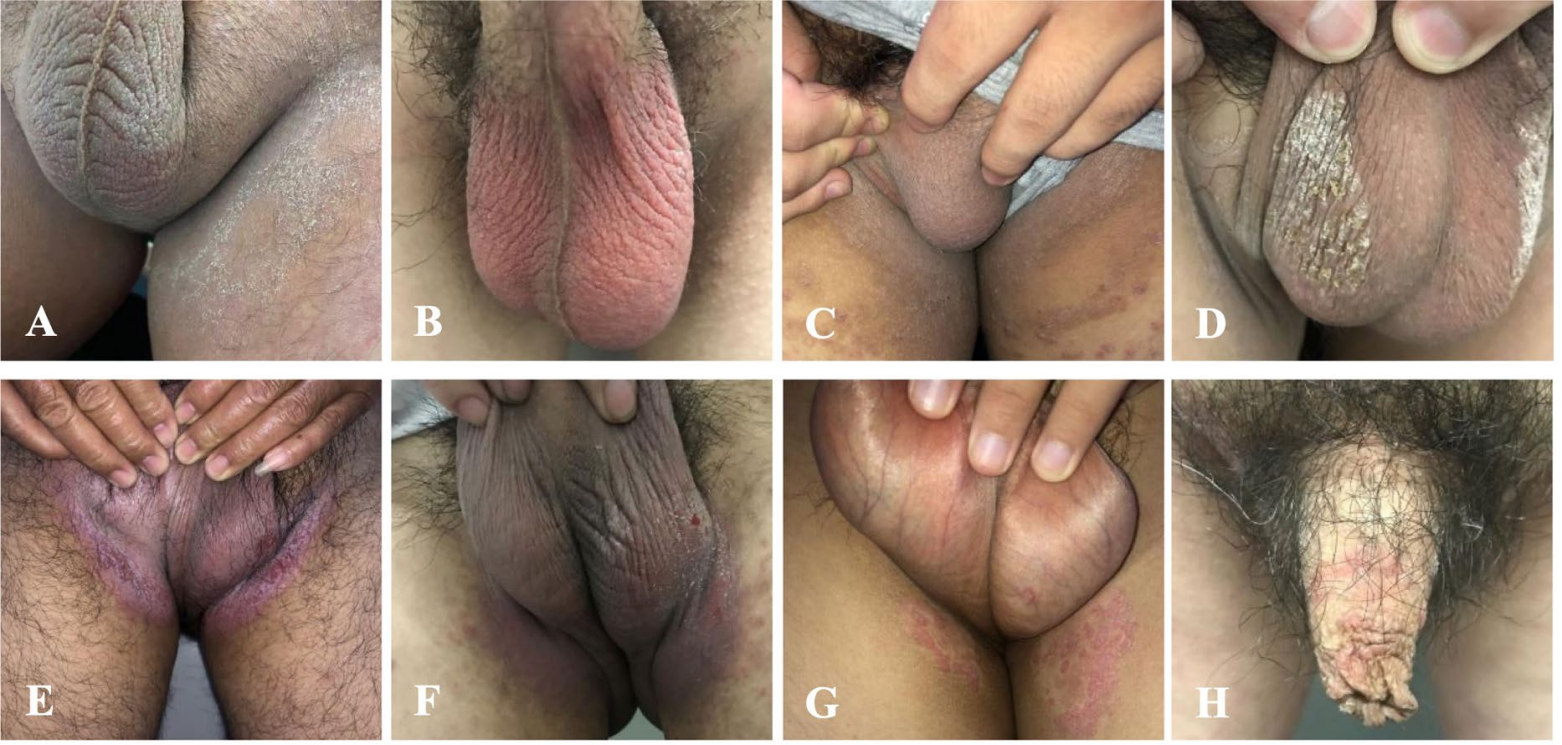
Clinical manifestation of male genital fungal infections. **A** erythematous, dry diffused whitish scaly lesions caused by *T. rubrum.* **B** erubescent complexion by *C. albicans*. **C** slightly diffused powdery scalings by *T. rubrum*. **D** yellowish scutular scales by *T. rubrum*. **E** erythematous erosion by *C. albicans*. **F** erythematous erosion with frictional lichenoid lesions by *C. albicans*. **G** erythematous annular scales by *N. gypsea*. **H** erythematous, dry scales with distinct border by *T. rubrum*

The clinical characteristics of 74 cases with fungal infections on scrotum could be roughly divided into six types. The most common clinical characteristic of tinea scrotum was erythematous, dry diffused whitish scaly lesions without a clear border, in 37 (50.0%) patients (Fig. 1a). Followed type, including 11 (14.9%) patients, presented as severely erubescent complexion with slight scaly lesions but itch seriously (Fig. 1b). Ten patients (13.5%) manifested as slightly diffused powdery scales with or not mild erythema (Fig. 1c), which was all noted for pruritus, and 9 out of 10 patients had tinea cruris in the meantime. Eight (10.8%) patients showed scutular scales with 2 whitish and 6 yellowish (Fig. 1d). Seven (9.5%) patients showed erythematous erosion, two of them with frictional lichenoid lesions (Fig. 1e-1f), which all initially extended from the groin. One (1.3%) showed erythematous annular scaly lesions (Fig. 1g) caused by *N. gypsea*. Most of the above manifestations had an obvious difference when compared with tinea corporis and cruris. The lesions such as erubescent complexion and erythematous erosion were caused by Mainly *C. albicans*, while most of others were caused by *T. rubrum.* There were 7 cases of penile fungal infection, 6 of which presented as annular erythematous, dry scales with distinct border (Fig. 1h) and one as several erythematous papules. The detailed correlations of clinical characteristics with pathogen spectrum were summarized in Table 2.

**Table 2:** The detailed correlations of clinical characteristics with pathogen spectrum in the 79 male patients with genital cutaneous fungal infections

Infection sites	Clinical characteristics	Etiology, case number					Total (%)
		*T. rubrum*	*C. albicans*	*N. gypsea*	*E. floccosum*	negative	
Scrotum	Erythematous, dry diffused whitish scaly lesions	29	5	0	0	3	37 (45.68)
	Erubescent complexion	2	8	0	0	1	11 (13.58)
	Slightly diffused powdery scalings	9	0	0	1	0	10 (12.35)
	scutular scales	6	0	1	0	1	8 (9.88)
	Erythematous erosion,two with lichenoid dermatitis	0	7	0	0	0	7(8.64)
	Erythematous annular scales	0	0	1	0	0	1 (1.23)
Penis	Erythematous, dry scales with distinct border	4	2	0	0	0	6 (7.41)
	Erythematous papules	1	0	0	0	0	1 (1.23)
Total (%)							81 (100)

### Predisposing Factors

Among these 79 patients, 74 (93.7%) complained of pruritus, and 61 (77.2%) had concurrent tinea cruris. Forty-one cases of these patients with tinea cruris carried out fungal culture, and positive results were found in 36 (87.8%) patients, and 34 of these 36 patients showed identical pathogens in groin and scrotum/penis, except two patients were identified as *C. albicans* in the scrotum but *T. rubrum* in the groin. Tinea pedis concomitantly appeared in 28 cases (35.4%), and multiple generalized ringworm of the whole body were found in 3 cases. Other underlying predisposing factors, such as topical corticosteroid agents (28 cases, 35.4%). Some patients used topical corticosteroid creams before visiting our hospital, however, the dosages used were unclear, and the applying times were arbitrary, mostly 1–4 weeks. In addition, excessive exercise and easy to sweat (26 cases, 32.9%), domestic pets (5 cases, 6.3%), wearing tight underwear frequently (4 cases, 5.1%). Only one patient had a history of diabetes, and all patients had no histories of using immunosuppressants recently.

### Treatment

Topical application of 2% miconazole nitrate cream and terbinafine hydrochloride gel were administrated for patients with local skin lesions and mild skin lesions (twice a day and lasting for 2–4 weeks); and itraconazole tablets or capsules were added for those patients with more serious skin lesions or complicated infection (orally, once a day, 0.2 g for each time, lasting for 2 weeks). One month later, through telephone follow-up, all patients reported significant improvement or cure in their skin lesions.

## Discussion

In this study, the clinical characteristics, pathogenic agents and predisposing factors of 79 cases patients with fungal infection on scrotum (74 cases) and penis (7 cases), including 2 cases infected on both scrotum and penis, were analyzed.

During the period for sample collection of the 79 cases, about 4000 male patients with inguinal fungal infections visited our department, which indicated the rarity of male genital fungal infections, particularly for the infection on penis (only 7 cases).

Among the 74 cases of cutaneous fungal infection on scrotum, 69 cases were positive for pathogens culture. The most common pathogenic agent was *T. rubrum* (46/69, 66.7%). A report coming from Siena and Terni (Italy) showed that 3 of 9 cases of tinea genitalis were found with scrotal lesions and isolated *T. rubrum* from 2 cases and *E. floccosum* from remained one case [6]. Another report from India showed that 10 of 24 cases of tinea genitalis were found with scrotal lesions and all identified isolates were *T. rubrum* [7]. A study coming from Hangzhou and Ningbo, Zhejiang Province, China obtained 63 positive isolates from 113 cases of tinea scrotum by culture, and 60 of which were *T. rubrum* (95.2%) and 3 were *N. gypsea* (4.8%) [15]. The results in these above three papers showed identical result with ours in the present study that *T. rubrum* was the main pathogen of cutaneous fungal infection on scrotum. However, Prohić et al. [8] reported 17 cases of tinea genitalis involved 13 cases of tinea scrotum, *Microsporum (M.) canis* was found in 10 (76.9%) patients, followed by *E. floccosum*, in 5 (38.5%) and *T. mentagrophytes var. interdigitalis* in 2 (15.4%) patients. The high frequency (90.4%) of *M. canis* in their population from Sarajevo area maybe the reason of high isolated rate of the species from genitalis. Another report from Guangzhou, Guangdong Province, China showed that *N. gypsea* was the most common species isolated in 17 of 35 (48.6%) cases of scrotal fungal infections, while *T. rubrum* was the secondly common isolates (9 of 35, 25.7%) [4]. The lesions caused by *N. gypsea* often presented as special manifestation such as crusts, which was more likely to attract the attention of doctors and was easily documented down. This is one possible reason for the high isolated rate of *N. gypsea*.

In our study, the most common clinical characteristics of scrotal lesions infected by *T. rubrum* were erythematous, dry diffused whitish scaly lesions, followed as slightly diffused powdery scales and scutular lesions. The first two types were described in most literatures [4, 6, 7, 15]. But the scutular scales caused by *T. rubrum* was only described in one paper from Hangzhou, China, which reported that10 cases of favus of scrotum were all infected by *T. rubrum* [5].

*C. albicans* was the second most common pathogen (20/69, 29.0%) in our study. However, there are less reports of scrotal skin fungal infection caused by yeast. Yin S et al. [4], reported that there were 8 (8/35, 22.9%) cutaneous yeast infections on scrotum including 3 cases of *C. albicans*. There was another case report about tinea scrotum by *C. guilliermondii* [9]. The presentation caused by *C. albicans* in our study was erubescent complexion erythematous erosion, lichenoid dermatitis and erythematous, dry diffused whitish scaly lesions. Eight of the 11 patients presenting erubescent complexion had ever applied topically corticosteroid agents on the groin and scrotum before visiting our department. The number of people who have used topical corticosteroid agents in the erubescent complexion group was significantly more than that in non-erubescent complexion group (8/11 versus 20/68; *P* < 0.05). Therefore, the erubescent complexion was possibly an allergic reaction. In the report from Yin S et al. [4] the clinical manifestations caused by yeasts was described as small annular erythema with eroded center, diffused powdery scales, and whitish dry scaly crusts, which is similar to the manifestations of our patients, although we did not find any scutular lesions. However, the clinical presentation by *C. guilliermondii* was also scutula-like lesion [9].

Only 2 (2.9%) species of *N. gypsea* were isolated in our study, one presented as yellowish scutular scales, which was the most common manifestation on scrotum caused by *N. gypsea*, the scutular lesions was sometimes described as pseudomembranous-like or white paint dot-like lesions in some reports [4, 10–12]. Scutula was usually the characteristic lesion of favus caused by *T. schoenleinii* [16] and tinea scrotum caused by *N. gypsea*, but now the scutular-skin lesions of scrotal scrotum caused by *T. rubrum* or yeast can also be found. Another one case caused by *N. gypsea* in our study manifested as several erythematous annular scaly lesions, which was identical to clinical manifestation from the patient's groin, and the course of disease was only 3 days. This type of scrotal lesion caused by *N. gypsea* has not been reported in other literatures. Possibly, the time of infection was too short to turn into scutula.

We only found 1 (1.4%) case of fungal infections on the scrotum caused by *E. floccosum*, which showed slightly diffused powdery scales. In the report from Yin S et al. [4]. there were 2 (5.7%) cases of scrotal fungal infection caused by *E. floccosum* and all presented as erythematous, slightly scaly lesions. Romano C et al. [6]. reported one case of scrotal fungal infection by *E. floccosum* also presented as erythematous, slightly scaly lesions.

Some reports identified that capric acid, one of the fatty acids in the epidermal barrier and particularly abundant in scrotal skin, might act as antifungal factor, and some fungistatic serum factor and sebum contributed to the low frequency of scrotal fungal infection [6, 8]. Liu ZH et al. [5] found some distinction in the level of hyphae penetrated the horny layer cells of scrotum stratum with that in groin stratum, and distinction in the hyphae structure on the two sites by scanning electron microscopy and transmission electron microscopy and speculated these distinctions might be the cause of the inconsistent appearance of lesions from tinea scrotum and tinea cruris. However, until now, the mechanism of mycelium invasion and immune resistance or chemical inhibition in scrotal fungal infection is still unclear.

In our study, there were only 7 patients with fungal infection on penis. The pathogenic isolates included 5 of *T. rubrum* and 2 of *C. albicans*. The clinical manifestations of 4 cases of the former and 2 cases of the latter all presented as annular erythematous and dry scales, the remained one case of the former showed erythematous papules. Romano C et al. [6] introduced that 5 out of 9 cases of tinea genitalis involved penis, 3 (60.00%) of which were caused by *T. rubrum* and all presented as scaly erythematous lesions. Another 2 with erythematous papules were caused by *T. mentagrophytes var. interdigitalis* and *E. floccosum*. Verma SB et al. [7] reported 24 cases of tinea penis with only 11 cases positive by culture and all of them were identified as *T. rubrum*, 8 of 24 cases of tinea penis presented as classical presentation of annular hyperpigmented scaly lesions and other cases manifested atypical presentations including pustules or thin powdery scalings and so on, the author suggested that the atypical types were due to the applying of various broad-spectrum steroid antifungal and antibacterial cream. Pielop J et al. [13] reported that 4 cases of penile tinea due to *T. rubrum* presented as annular erythematous plaque with fine silvery scale.

The common predisposing factors including concomitant fungal infection such as tinea cruris (77.2%), tinea pedis (35.4%), topical corticosteroid agents (35.4%) and high moisture (32.9%) were found in our study. Si Z et al. [15] reported that the concurrent infection rate of tinea cruris and tinea pedis in 113 cases of scrotum were respectively 89.38 and 46.01%. In the report from India [7], all 24 cases of patients gave history of applying different brands of triple and quadruple combination creams containing clobetasol propionate, beclomethasone, antifungals and antibacterial agents. In some reports, inadequate hygiene, occlusion and diabetes were also common factors [7, 8, 13].

There are some limitations of the present study. Onychomycosis was not recorded. Most pathogens of concurrent fungal infection at other sites were not cultured and identified.

In summary, the cutaneous fungal infection on scrotum presented various atypical or slight clinical manifestations that could be easily overlooked. Any marks/changes on the scrotum should be taken seriously, especially in patients with tinea cruris.

## Data Availability

The original contributions presented in this study are included in the article. Further inquiries can be directed to the corresponding authors.
